# The Achilles’ heel of cancer: targeting tumors via lysosome-induced immunogenic cell death

**DOI:** 10.1038/s41419-022-04912-8

**Published:** 2022-05-30

**Authors:** Taritsa Iulianna, Neote Kuldeep, Fossel Eric

**Affiliations:** 1 Odin Therapeutics, Newton, MA 02458 USA; 2 Feinberg School of Medicine, Northwestern University, Chicago, IL 60611 USA

**Keywords:** Cancer immunotherapy, Immunotherapy, Tumour immunology, Apoptosis, Adaptive immunity

## Abstract

Interest in the lysosome’s potential role in anticancer therapies has recently been appreciated in the field of immuno-oncology. Targeting lysosomes triggers apoptotic pathways, inhibits cytoprotective autophagy, and activates a unique form of apoptosis known as immunogenic cell death (ICD). This mechanism stimulates a local and systemic immune response against dead-cell antigens. Stressors that can lead to ICD include an abundance of ROS which induce lysosome membrane permeability (LMP). Dying cells express markers that activate immune cells. Dendritic cells engulf the dying cell and then present the cell’s neoantigens to T cells. The discovery of ICD-inducing agents is important due to their potential to trigger autoimmunity. In this review, we discuss the various mechanisms of activating lysosome-induced cell death in cancer cells specifically and the strategies that current laboratories are using to selectively promote LMP in tumors.

## Facts

- The lysosome plays a central role in regulated cell death and the extent of its membrane permeabilization can determine the route of cellular demise that follows cellular stress, including whether immunogenic cell death occurs.
- Immunogenic cell death can be measured via several damage-associated molecular patterns. Some of the most notable examples being ATP, CALR, HSP70 and 90, and HMGB1, which are now considered hallmark biomarkers of this mechanism, and can all be linked back to actions at the lysosome.
- Reactive oxygen species can either be generated intrinsically or extrinsically to the cell. Well-studied cancer drugs known to induce immunogenic cell death almost exclusively generate ROS intrinsically, though this pathway depends on several factors that make some cancer cells resistant to such cell killing.

## Open questions

- Can an understanding of intrinsic vs. extrinsic generation of reactive oxygen species help researchers better target and kill cancer cells that were previously thought to be resistant to traditional therapies?
- How can we develop cancer therapies that specifically target the lysosome and promote long-term immunity through immunogenic priming to cancer antigens?

## Lysosome characteristics, function, and alteration in cancer

### Characteristics

Fundamentally, the lysosome is a ubiquitous intracellular organelle often compared to a digestive sac. In fact, the term *lysosome* originated from the Greek words meaning destructive or dissolving body. Structurally they appear as dense spheres or tubes generally less than one micron across, though the size and number are variable depending on the amount of unprocessed cellular debris that has been taken up. The organelle has a single phospholipid bilayer membrane and an acidic (pH 4.5) vesicular space filled with degradative enzymes known as hydrolases as well as specific membrane proteins. To differentiate them from late endosomes, it is of note to mention that lysosomes lack the mannose-6-phosphate receptor (M6PR). There are 25 or more unique membrane proteins residing throughout the lipid bilayer. The most abundant by far are lysosome-associated membrane protein 1 (LAMP-1) and lysosome-associated membrane protein 2 (LAMP-2) which together make up over half of the total membrane proteins. LAMP-1 is involved in cell-cell adhesion and migration and LAMP-2A has been identified as a receptor for chaperone-mediated autophagy (CMA) [1]. Both LAMP-1 and LAMP-2, along with lysosomal integral membrane protein 2 (LIMP2), CD63, and molecular chaperone heat shock protein 70 (HSP70), work to trap the resident hydrolases inside the lumen [2].

Besides the membrane proteins, the second class of proteins essential for lysosomal function are the hydrolases. There are over 60 hydrolases, including proteases, peptidases, phosphatases, nucleases, glycosidases, sulfatases, and lipases, that have been identified and characterized [3]. Each targets a specific substrate and together this class of proteins can digest a broad spectrum of macromolecules. Among the hydrolases, most studied and relevant are the cathepsin family of proteases which break down into three subtypes depending on which amino acid residue is found in the active site. The serine group includes cathepsins A and G, cysteine for cathepsins B, C, F, H, K, L, O, S, V, W, and X, and aspartic acid in cathepsins D and E.

### Function

Lysosomes play a crucial role in many cell processes, ranging from protein secretion to cell signaling. Notably, the lysosome is the mediating organelle for endocytosis, phagocytosis, and autophagy and acts as the end site of the cellular degradative pathway. Their involvement in degradation is two-way since lysosomes both receive extracellular material through the endocytic pathway or intracellular material through autophagy, as well as fuse with other plasma membranes before secreting their acidic contents. It is this bimodal trafficking property that makes lysosomes highly dynamic [1]. In the endocytic pathway, exogenous material enters the cell as an early endosome via an internalized plasma membrane. Over the course of roughly forty minutes, the early endosome matures into a late endosome and eventually into a lysosome. The full maturation process involves changes in morphology, an exchange of membrane components and the acquisition of lysosomal components, the formation of intraluminal vesicles, and a significant decrease in pH to accommodate for the function of lysosomal hydrolases [3].

Autophagy can be categorized into three subtypes: chaperone-mediated, microautophagy, and macroautophagy. In the chaperone-mediated process, specific cytosolic proteins distinguished by motifs on their surface are delivered to the receptor LAMP-2A on the lysosomes via chaperone proteins. Microautophagy is the non-selective and direct engulfment of cytoplasmic cargo at a boundary membrane. Macroautophagy is considered the major subtype and involves the formation of an autophagosome from a small amount of cytoplasm with soluble materials and potentially organelles inside a double membrane. This vesicle then fuses with the lysosome becoming an autolysosome to be degraded. The lysosomal hydrolases break down the cargo inside the vesicle and in this way, damaged or unneeded cellular material can be recycled.

Lysosomes have also distinguished themselves as key to cell death signaling. They mediate the processes of cellular apoptosis and necrosis through a process called lysosomal membrane permeabilization (LMP). Various stimuli can trigger the lysosomal membrane to degrade, such as p53 signaling [4–6], oxidative stress [7], and proteases (caspases, cathepsins, and calpains) [8–11]. Once the lysosome’s membrane is damaged, then cathepsins like B and D, other hydrolases, and lysosomotropic detergents contained within the lysosome spill out into the cell’s cytoplasm and initiate cell death [3]. The currently proposed model is a positive quantitative relationship between the level of lysosomal hydrolases in the cytosol (e.g., cysteine cathepsins and β-N-acetyl-glucosaminidase (NAG)) and the extent of LMP [12–14]. Regulated and partial leakage of the destructive lysosomal hydrolases can lead to apoptosis while massive leakage or total rupture triggers necrosis [12]. The distinction between LMP-induced apoptosis versus necrosis is noted as far back as studies in 1997 where cells exposed to toxic irradiation for four minutes exhibited moderate cathepsin D release and became apoptotic, while those irradiated for eight minutes were seen to have massive amounts of hydrolase leakage, severe lysosomal damage, and ultimately necrosis [3].

To combat against cell-damaging agents, some proteins protect against lysosomal membrane leakage. These include LAMP-1, LAMP-2, and heat shock protein 70 (Hsp70) [15–18]. These proteins, as discussed previously, are crucial for stabilizing the membrane and trapping hydrolases inside the acidic environment of the lysosome. The demonstrated effects that lysosomal leakage has on cell death allude to the intriguing and powerful link between lysosomal membrane permeabilization and apoptotic cell death mechanisms.

### Alterations in cancer

In cancer, cells have been found to have mutations in apoptotic mechanisms that protect them from being killed [1]. Specifically, the autophagy-lysosome pathway is intricately involved in cancer’s mechanism of escaping immune detection and apoptosis. Changes in lysosomal morphology have been reported in all types of cancer. The lysosomal membrane protein acid sphingomyelinase (ASM) has a downregulated expression in gastrointestinal, hepatocellular, salivary gland, renal, and head and neck carcinomas [19]. The destabilized lysosomal membrane in malignant cells predisposes them to the apoptotic cascade following the leakage of degradative enzymes. The lysosomes in tumor cells can also look dramatically different on light microscopy: they are larger in size and are differently distributed within the cell compared to non-malignant cells. For example, as compared to non-malignant breast epithelial cells, oncogene-associated MCF-10A-neoT cells had lysosomal distribution predominantly in the cell periphery rather than the perinuclear region [20].

Changes in lysosomal function have been reported in all types of cancer. Disease progression involves an increase in the levels of lysosomal enzymes. Cathepsins are frequently overexpressed in tumors—for example, cathepsin B has been found to be higher in colorectal cancer cells and cathepsin D is increased in melanoma, glioma, and lung cancer [21–23].

Both the morphological and enzymatic changes to the lysosome in cancer cells may grant them selective advantages such as better angiogenesis, drug resistance, and invasive growth. These theorized effects were specifically demonstrated in Yanamandra’s 2004 study of human glioblastoma, where blocking cathepsin B expression suppressed angiogenesis in these cancer cells [24]. Also, the crucial role of cysteine cathepsins B, L, and S in neovascularization in pancreatic cancer has been described [25, 26]. While these changes to the lysosome may grant some advantages to the tumor cell, the elevated levels in damaging cathepsin activity and decreased levels of stabilizing lysosomal membrane protein may sensitize the tumor cells to LMP-initiated cell death. The differences in lysosome composition recently have proven to be mechanisms for exploitation in lysosomal anticancer therapies that take advantage of tumor cells being more sensitive to LMP [27].

## Immunogenic cell death

### Features of regulated cell death and immunogenic cell death

The lysosome’s role in cell death is of interest due to its connection with autoimmunity against cancer cells. When cells die, it was traditionally believed that they could undergo one of two main types of cell death: apoptosis which is programmed and regulated cell death that was believed to be non-immunogenic; or necrosis which was believed to be the result of accidental events induced by different stimuli such as osmotic conditions, a lack of nutrient supply and characterized by an inflammatory and immunogenic response. Advancements in biochemistry have allowed for the recent amendment of this formerly simplistic understanding of cell death. The Nomenclature Committee on Cell Death (NCCD) has now added and described twelve classes of programmed cell death which are based on differences in molecular mechanisms, morphological features, and immunomodulatory profiles [28]. The current twelve classes of regulated cell death defined by the NCCD are intrinsic apoptosis, extrinsic apoptosis, mitochondrial permeability transition (MPT)-driven necrosis, necroptosis, ferroptosis, pyroptosis, parthanatos, entotic cell death, NETotic cell death, autophagy-dependent cell death, immunogenic cell death, and lysosome-dependent cell death. We will briefly describe some of the features of these types of cell death, though recognize the interconnectivity between the different forms. There is a fine distinction to be made between apoptotic ICD from necroptotic ICD from ferroptosis and pyroptosis [29]. Each have unique molecular characteristics and one method of distinguishing each from each other is by analyzing how each incites immunogenicity following the destruction of the cell.

First, apoptosis is one form of regulated cell death which involves controlled leakage of lysosomal hydrolases, chromatin condensation, nuclear fragmentation, cell shrinkage, and the sequestration of the cytoplasm into vacuoles to be cleared by autophagy. Under certain conditions, a certain type of cell demise with unique characteristics may ensue called immunogenic cell death (ICD). Here, the pathway of death stimulates a local or systemic immune response against dead-cell antigens. Apoptotic ICD is associated with an organized intracellular degradation of the cell that allows for controlled immunological clearance and disposal [28].

In contrast, necroptosis is a form of necrosis very different from accidental necrosis, and is induced by massive leakages of lysosomal hydrolases, cell swelling, and later plasma membrane blebbing [30]. It is often linked to ICD. While previously it had been assumed that ICD was more commonly seen following necrosis rather than apoptosis, more recent investigations have shown that pairing apoptosis with tolerogenicity and necroptosis to immunogenicity is overly simplified and not always correct [31]. Types of apoptosis that can spur an immunogenic response and necrotic events that lead to immunosuppression have been documented. Necroptosis can be understood as a backup cell death mechanism that occurs when extrinsic factors fail to initiate apoptosis due to pharmacological or genetic inhibitors. Necroptotic ICD is initiated by a specific set of activated surface-associated death receptors (e.g., tumor necrosis factor receptor 1 (TNFR1), DR4/5, FAS receptor) [32, 33]. Necroptosis is further differentiated from apoptotic ICD in that it causes necrosis-associated inflammation.

Ferroptosis is initiated by specific iron-dependent physiologic triggers such as high extracellular glutamate, cysteine deprivation, or other conditions where high levels of poly-unsaturated fatty acids are incorporated into a cell membrane [29]. The intracellular iron required for ferroptosis induces reactive oxygen species production and lipid peroxidation [34]. The functional role of this cell death pathway is believed to be protective because of its systemic removal of cells that produce excessive amounts of electrophilic intermediates [29]. It has been linked to immunogenic cell death due to the similar downstream characteristics that cells undergoing ferroptosis present compared to those undergoing apoptotic and necroptotic ICD [35]. Finally, pyroptosis is an inflammatory type of regulated cell death initiated either by gasdermin D or gasdermin E, each cleaved to its active form by caspase 1/4/5/11 or caspase 3, respectively, after these enzymes are triggered by reactive oxygen species [36, 37]. Immunogenic cell death is the result after pyroptosis-associated gasdermin action begins a cascade of DNA breakage, chromatin condensation, cell swelling, rupture, and the final release of cell contents. There may be crosstalk between apoptotis, necroptosis, ferroptosis, and pyroptosis, and these have been discussed elsewhere [29].

While these forms of regulated cell death all differ in the way they trigger the process of ICD, they converge on a result which is functionally the same. Immunogenic cell death can be seen as the final downstream consequence of variable molecular events that give rise to recognition mechanisms at the level of dendritic cells—the first innate immune responders in ICD. To distinguish when immunogenicity is emerging, several parameters have been defined. First, ICD is obligatorily preceded by two forms of premortem intracellular stress: endoplasmic reticulum stress and autophagy. It is widely accepted that an ICD inducer be capable of promoting reactive oxygen species-based ER stress [38]. Stress conditions produce an accumulation of reactive oxygen species (ROS) and misfolded proteins which causes the endoplasmic reticulum to activate a complex signaling pathways network, called the unfolded protein response (UPR). Three different sensors on the ER-membrane can initiate the UPR signaling pathway: protein kinase R-like endoplasmic reticulum kinase (PERK), activating transcription factor-6 (ATF6) and inositol-requiring transmembrane kinase/endoribonuclease 1 (IRE1) [39, 40]. PERK triggers the phosphorylation of α-subunit of eukaryotic initiation factor 2 (eIF2α), leading to cell cycle arrest, and apoptosis-signaling pathway [41]. ATF6 when activated by ROS and large numbers of unfolded proteins translocates to the Golgi apparatus then the nucleus to upregulate other proteins required for the UPR. IRE1 activates X-box-binding protein 1 (XBP1), which further induces the expression of UPR stress signals, including tumor necrosis factor receptor-associated factor 2 (TRAF2), apoptotic-signaling kinase-1 (ASK1), Jun-N-terminal kinase (JNK), and p38 mitogen-activated protein kinase (MAPK) [39, 40, 42].

These stress signals further increase ROS levels inside the cell and cause the early translocation of calreticulin (CALR), an ER luminal resident protein, to become translocated to the outer leaflet of the dying cell’s plasma membrane. ATP is secreted, and as the cell membrane becomes permeabilized, there is the release and exposure of heat shock proteins (HSP70 and 90) and the nuclear protein HMGB1. Once on the outside of the cell membrane, they bind to CD91, P2RX7, and TLR4. ATP and the heat shock proteins stimulate the recruitment of dendritic cells to the site of immunogenic cell death, calreticulin facilitates the engulfment of dead-cell antigens by DCs [43], and HMGB1 primes the antigens for presentation to T lymphocytes. The culmination of these events is the production of a potent cytokine-mediated immune response (primarily IL-1β, IL-17, and IFN-γ) to eradicate the population of dying cells [44, 45]. Due to their extracellular action to mediate ICD, the levels of ATP, CALR, HSP70 and 90, and HMGB1 are now hallmark biomarkers of this mechanism [46]. Collectively, this group of biomarkers are known as damage-associated molecular patterns (DAMPs) and their concomitant presence implies immunogenic cell death.

When induced, immunogenic cell death allows the body to overcome the immunosuppressive phenotype of the tumor microenvironment. This is done by facilitating interaction between dendritic cells and T cells to activate an immunogenic T-cell response. The DC-T-cell interaction consists of three signals: antigen presentation, co-stimulation, and the generation of specific cytokines [47]. Antigenicity and adjuvanticity are important aspects of ICD. Expression of DAMPs attracts dendritic cells to engulf the stressed cell. As the dendritic cells engulf fragments of the dying cell, it incorporates antigenic peptides into MHCs, and this process is termed antigenicity. Adjuvanticity is the optimized and potent activation of T cells which is facilitated by the maturation signals created by DAMPs. Once activated, the body’s T cells can seek out and kill any cancer cell expressing the same antigen signature. Thus, ICD allows for precise and antigen-specific clearance of malignant cells. This specific quality makes it a highly attractive target for cancer therapeutics.

### Role of the lysosome

It is the action of the lysosome that seems to underpin many of the initiatory events responsible for triggering highly specific dead-cell immune responses. The lysosome’s role is similar in each form of regulated cell death linked to ICD, including the apoptotic, necroptotic, ferroptotic, and pyroptotic forms. Each type of premortem stress that leads to ICD involves the generation of reactive oxygen species which can either be internally or externally generated. The abundance of ROS, once sensed by the cell, induces lysosome membrane permeability [48]. The exact route by which reactive oxygen species triggers LMP differs depending on the mechanism of lysosome-induced cell death, and these routes will be discussed in depth later in this review. Once LMP begins, it triggers a downstream pathway involving many more ICD-specific biomarkers. Lysosomal hydrolase Cathepsin D along with the zymogen form of caspase 8 are released into the cytoplasm [46]. While there are two distinct pathways for the activation of caspase 8 as reviewed by Cohen et al., the most direct involves the activation of caspase 8 by Cathepsin D and this activation is demonstrated to be crucial to the initial steps of cell death [49, 50].

**Fig. 1:**
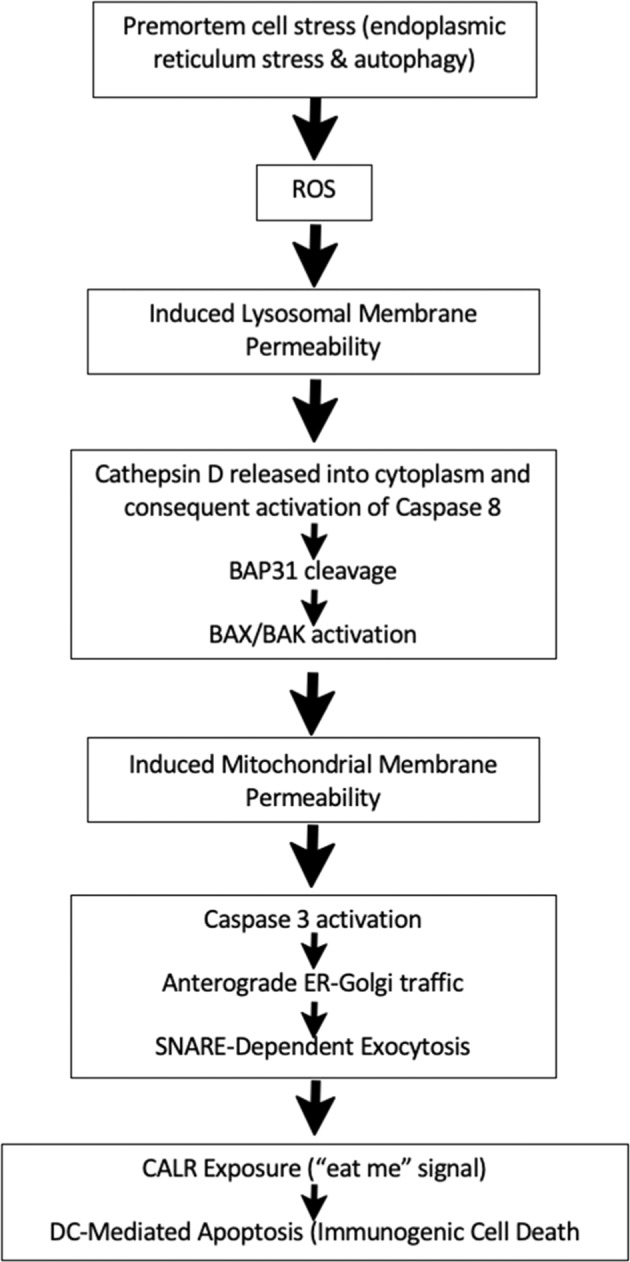
General pathway of ICD. Both non-extrinsic and intrinsic mechanisms are delineated.

Once cleaved to its active form, caspase 8 induces ER-associated BAP31 cleavage through downstream caspase activation [51]. Caspase 8 also cleaves the BH3-only protein Bid into its truncated form, t-Bid, which moves to the outer mitochondrial membrane [52]. Both BAP31 and Bid cleavage in turn stimulate oligomerization of BAX or BAK, two pro-apoptotic enzymes belonging to the BCL-2 family which puncture the outer mitochondrial membrane [51, 53]. BAX/BAK thus induce mitochondrial outer membrane permeabilization which accelerates the rate of cell death by activating caspase 3, cytochrome *c* and other degradative enzymes [51], anterograde ER-Golgi traffic, SNARE-dependent exocytosis, and then the exposure of CALR on the cell surface [46] (Fig. 1). Many of these protein levels (caspase 8, BAP31, BAX/BAK, etc.) can be measured intracellularly before calreticulin appears on the surface of the cell as an even earlier marker of ICD [46].

Lysosomal membrane permeabilization seems to be central for apoptotic or necrotic cell death. It appears that the degree of lysosomal membrane leakage regulates an apoptotic versus necroptotic response [12]. A more limited, controlled LMP causes apoptosis and an induction of immunogenic cell death, while a total leakage induces necrosis. The lysosome has also been implicated as a crucial part of ferroptosis by Torii and colleagues, who found that inhibiting lysosomal action also repressed ferroptosis by reducing basal ROS generation and blocking iron metabolism in ferroptosis-sensitive cells [34]. Pyroptosis is also dependent on the lysosome, as inducers of pyroptosis require caspase-1 to act on lysosomes and promote the organelles’ leakage for eventual cell death [54].

We propose an alternative mechanism for understanding ICD that underlines the central role of the lysosome in initiating immunogenic cell death. We will term the entire process stemming from LMP as “lysosome-induced immunogenic cell death,” or LIICD, to highlight the necessary role the lysosome plays in this apparatus.

Since cancer cells have weaker lysosomal membranes as compared to their non-diseased counterparts, they are selectively sensitive to cell death—a fact which makes the lysosome a prime target for emerging anticancer therapeutics. However, among the types of lysosome-targeting drugs currently being developed, there is observed drug resistance. This is due to the fact that LIICD works through two routes, an extrinsic and intrinsic pathway. To understand why some tumors remain “cold” (or unresponsive) to lysosome-targeting drugs [55], it is important to note the differences in the two pathways. Only targeting the intrinsic pathway, which can be mutated in cancer cells, may make certain drugs ineffective treatments. A more detailed view of LIICD is necessary to understand how to strike cancer at its Achilles’ heel: to kill the tumor, eradicate drug resistance, and prime the body to recognize and eradicate diseased cells should they be reintroduced.

## The intrinsic pathway of LIICD: hitting “hot” tumors

### Mechanism of action: intrinsic generation of reactive oxygen species

Previous literature has differentiated ICD into two different types, either Type I or Type II [56]. These varieties are distinguished based on whether the inducers of immunogenic cell death are acting at the nucleus level or at the endoplasmic reticulum. We will refer to type I as an “intrinsic” mechanism while type II is “extrinsic.” The intrinsic versus extrinsic distinction in lysosome-induced cell death refers to where the superoxides are produced that will trigger downstream lysosome membrane permeabilization. We will begin by detailing type I, or intrinsic, LIICD. In the intrinsic pathway, the process of LIICD is activated by the intracellular production of reactive oxygen species. This is unlike the extrinsic type II variety of cell death where there is an extrinsic source of ROS which interacts with the cell to induce LIICD. The intrinsic cascade is classically documented in Polyak et al.’s work from 1997 and has since been targeted in many anticancer drugs [57].

Temporally, the intrinsic process begins with cytotoxic agents such as anthracyclines, UV and ionizing radiation, quinone compounds, and some inflammatory cytokines which damage DNA. The mechanism of causing DNA damage varies with the type of oxidative stress. One example is the anthracycline doxorubicin which is speculated to intercalate into DNA and thereby disrupt the normal helical structure [58]. Damage to the cell’s genetic material activates the tumor suppressor gene p53. p53 is a sequence-specific DNA binding protein that plays a crucial role in regulating transcription and cellular responses under stress. p53 activation spurs expression of PIG3, a class of p53-induced genes which encodes for ROS-generating enzymes like NADPH oxidases [59]. NADPH-quinone oxidoreductase is a potent generator of reactive oxygen species.

**Fig. 2:**
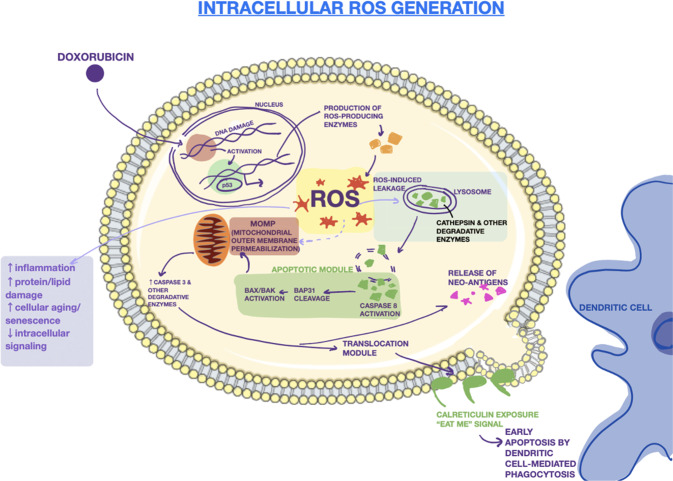
Intrinsic pathway of LIICD. The mechanism of induction of lysosome-induced immunogenic cell death is shown including reactive oxygen (ROS)-induced leakage of lysosomal membranes and mitochondrial outer membrane permeabilization (MOMP). These are followed by an increase in intracellular levels of degradative enzymes and the translocation of calreticulin (an “eat me signal”) to the cell surface.

Once in the cytoplasm, ROS immediately sets off a wide range of pro-apoptotic processes, including protein and lipid damage, inflammation, and mitochondrial outer membrane permeabilization. The intrinsically produced ROS is crucial to degrading the lysosomal membrane for LMP. The lysosome is one of the most sensitive organelles to ROS due to its lack of common antioxidant enzymes (such as superoxide dismutase, catalase, or glutathione peroxidase). Thus, when the levels of ROS are high, superoxide can easily cross through and damage the lysosomal membrane [60]. ROS destabilizes the lysosomal membrane via massive peroxidation of lysosomal membrane lipids [61]. Once the lysosome is permeable, its degradative contents leak out, initiating the final steps of LIICD. Experiments have shown that LMP allows for cathepsins to be rapidly released from inside the lysosome. More ROS is also generated from photosensitizers located in the lysosome [62]. The increased cathepsin levels and ROS within the cytosol activates effectors like BAX/BAK [60]. BAX/BAK activation and downstream dysregulated mitochondrial electron transport activity further destabilizes lysosome and mitochondrial membranes. The BCL-2 family members BAX/BAK increase the levels of cellular stress in the cell by creating pores in the outer mitochondrial membrane which allow for soluble proteins from the inner mitochondrial membrane space to escape. These mitochondrial proteins include, but are not limited to, cytochrome *c*, somatic (CYCS), which normally acts as an electron shuttle in the mitochondrial respiratory chain, and second mitochondrial activator of caspases (SMAC) [28, 63, 64]. Together with apoptotic peptidase activating factor 1 (APAF1) and pro-caspase 9 (CASP9), CYCS forms a complex called an apoptosome which activates caspases 3, 7, and 9 [28]. This produces a pro-apoptotic feedback loop where the caspases activate more BAX/BAK. BAX and BAK, dependent on BH3-only proteins, have also been shown to act back on the lysosomal membrane via destabilizing effects of the apoptosome, which damages the lysosome further [65–67]. The entire process of the intrinsic pathway is detailed in Fig. 2.

### Targeting the intrinsic pathway

Since its mechanism of action was first outlined, the intrinsic pathway of LIICD has been a popular target for several lysosome-acting cancer therapies. Inherent to all of these strategies is the induction of DNA damage that triggers a p53-dependent apoptotic response [68].

#### Photodynamic therapy (PDT) and UV radiation

One strategy to initiate LMP in cancer cells is to administer a photosensitizing agent followed by irradiation of the activated cells with a wavelength corresponding to the absorbance band of the sensitizer. One of the first clinically approved photosensitizers for cancer therapy was porfimer sodium, a water-soluble mixture of porphyrins, shown to be effective when combined with light irradiation at 630 nm at treating lung, esophagus, bile duct, bladder, brain, and ovarian cancer [69]. Other studies treated ex vivo cancer cell lines with photodynamic therapy and then re-implanted these cells subcutaneously into syngeneic immunocompetent mice to function as cancer vaccines most effective for early-stage tumors [56, 69]. Mechanistically, the process relies on the accumulation of the photosensitizers in the lysosomes. However, this strategy does not target ER stress directly. Rather it induces apoptotic cell death through DNA-replication proteins and stimulates ICD-associated immunogenicity through secondary or collateral stress effects [56]. Also, its effects are very limited to where the light sources can penetrate. While this is attractive for its decreased chance of off-target effects, it also makes it ineffective at treating metastatic cancers.

One study that further supports the limited effectiveness of PDT used low-density lipoprotein (LDL) treated with the photosensitizer 8-Methoxypsoralen (8-MOP) and UV light. The photosensitization technique was able to kill HuT-78 cells, a cutaneous T-cell lymphoma cell line, however, the cytotoxicity was less potent than with treating the cells with chemically peroxidized (via hydrogen peroxide and peroxidase) LDL. The difference in effectiveness of these treatments is believed to be because PDT works via the intrinsic method while chemically induced p-LDL affects the extrinsic LIICD pathway. Since HuT-78 cells have mutant p53 status, intrinsic LIICD showed less cytotoxicity [70].

Despite their limited effectiveness, photodynamic ICD inducers have been popular avenues for cancer therapeutic development. Thus, a great number of photosensitizers are currently used to assist in immunotherapy and the most common can be generally grouped into organic photosensitizers, inorganic photosensitizers, and organic–inorganic hybrid materials. The organic group includes indocyanine green (ICG) [71], chlorin e6 (Ce6) [72, 73], and pheophorbide A (PPa) [74]. These organic photosensitizers have struggled to prove efficient production of ROS in the cell, with weak effects on the lysosome. The inorganic group includes black phosphorus quantum dots (BPQDs) and black phosphorus nanovesicles (BPNVs) [75]. While this group has proven to be more efficient in generating ROS, their applications are limited due to their small size which causes quick clearance out of the body and limited accumulation in organelles. The organic–inorganic group of photosensitizers mainly consist of metal-organic frameworks of chlorin and chlorin derivatives like 5,10,15,20-tetra(*p*-benzoato)chlorin (H_4_TBC) [76]. Designed against colorectal cancer, these compounds have induced ICD locally though not systemically and have not been shown to generate a response against metastatic cancers. Porphyrin-based metal frameworks are also being explored [77]. The dependence on intrinsic production of reactive oxygen species in each category of photodynamic therapy greatly restrict the use of these therapies.

#### Anthracyclines

Anthracyclines, such as doxorubicin, have been used to induce LIICD through the intrinsic pathway. These compounds have been widely and definitively shown to induce immunogenic cell death since their administration causes cell death with ectopic expression of calreticulin, release of extracellular ATP as well as HMGB1 release—the hallmarks of LIICD [78, 79]. Since its effectiveness at sparking LIICD was established, anthracyclines have been used in mouse models of vaccination for colon carcinoma with successful long-term antitumor immunity [78].

The anthracyclines’ primary mechanism of action is the intercalation into DNA, which interferes with DNA replication and activates the p53-dependent generation of intracellular ROS [80]. Its accumulation in the nucleus allows it to induce LIICD but the production of reactive oxygen species is neither a primary effect nor is it specifically directed against the endoplasmic reticulum [80]. Thus, in cells where any of the genes are mutated that are necessary for anthracyclines’ induction of LIICD (e.g., p53, caspase, BAX, etc.), these drugs will be ineffective at causing the appropriate cytotoxicity of tumor cells and anticancer immune response. For example, doxorubicin’s failure to kill breast cancer cells where p53 is mutated is seen in recent work in the field by Fossel et al. [81]. When anthracyclines do not reach the nucleus because of defective subcellular localization (even if they are present in extranuclear organelles), this is yet another reason they may fail to cause cell death [82]. Moreover, it has been speculated that doxorubicin may enhance cancer therapy resistance due to its ability to upregulate lysosomal drug sequestration without appropriate LMP^2^. For all of these reasons, the probability of LIICD-resistant cancer variants forming increases when using anthracyclines as a therapeutic agent.

#### Mitoxantrone and oxaliplatin

Mitoxantrone (MTX) and oxaliplatin have been used in a fashion very similar to the anthracyclines to attack cancer cells and prime an anticancer immune response. Oxaliplatin is a platinum-based compound [83] and mitoxantrone falls under the class of drugs known as anthracenediones or anthraquinones [84]. These LIICD inducers also accumulate in the nucleus and act on the intrinsic pathway of immunogenic cell death. Oxaliplatin works as a coordination complex to inhibit DNA synthesis, while mitoxantrone inhibits topoisomerase II activity which again inhibits DNA synthesis and repair [80]. Both activate p53 to stimulate immunogenic cell death, though the effect is not direct. Rather, ER stress is a collateral effect [56]. These drugs have been used in osteosarcoma cell lines with the desired effects of inducing ecto-CALR expression, ATP release, as well as HMGB1 release [83].

Like the anthracyclines and PDT strategies, however, there have been serious drawbacks to MTX and oxaliplatin as anticancer therapies. The tumor-specific immune response has been compromised in cases where p53, BAX, and/or caspase 8 is mutated, as well as when autophagy-related genes needed for MTX/oxaliplatin’s efficacy, ATG5, ATG7, and BECN1 are inhibited [85, 86]. These have been linked to the failure to generate extracellular ATP after apoptosis and thus lack of a subsequent immune response in vivo. Also, these drugs (as well as PDT) rely on eIF2α phosphorylation [87, 88]. eIF2α, as discussed earlier, gets phosphorylated after PERK activation in the UPR signaling pathway which occurs with ER stress to induce autophagy. Some malignant cells lack this mechanism of PERK action on eIF2α, while others have constitutive attenuation of eIF2α phosphorylation—either of which create therapy-resistant phenotypes to resist the LIICD effects of both the mitoxantrone/oxaliplatin drugs as well as photodynamic therapy [89].

### Limitations of intrinsic LIICD-targeting therapeutics

While drugs that attempt to kill cancer cells and prime a memory response via the intrinsic pathway of lysosome-induced immunogenic cell death were initially promising due to their early validation of induced ICD markers, they only serve to treat a small subset of tumors known as “hot” tumors. That is, these drugs work against cancer cells with normal p53, BAX, caspase 8, and other apoptotic genes. Many of these types of tumor cells were not chemotherapy-resistant in the first place.

The global limitation of targeting the intrinsic pathway is that the p53 tumor suppressor gene is mutated in up to half of human tumors [90], and mutations in other genes necessary for controlled cell death may render even more cancer cells resistant to these ICD therapies. Drug-resistant cancers, or “cold” tumors, must be targeted via another route—one that bypasses the genome and acts directly at the level of ER stress to generate lysosomal membrane permeabilization [68]. This is why it is important to understand the extrinsic pathway of LIICD and identify drugs that induce immunogenic cell death via p53-independent, focused ROS-based ER stress.

## The extrinsic pathway of LIICD: how to target “cold” tumors

### Mechanism of action: extrinsic generation of reactive oxygen species

**Fig. 3:**
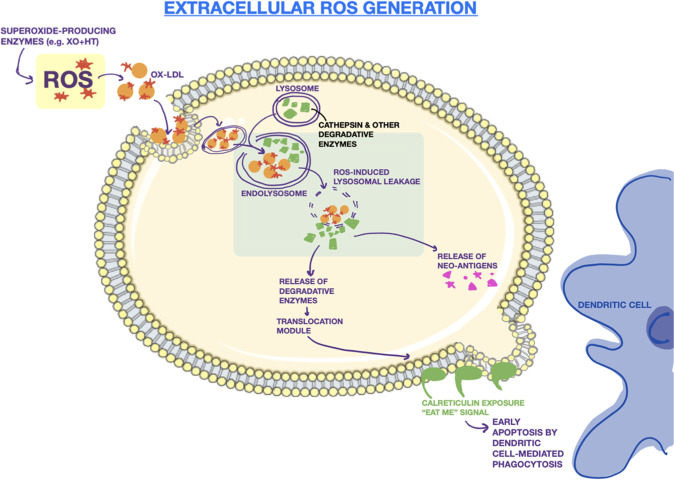
Extrinsic pathway of LIICD. The mechanism of extrinsically-generated reactive oxygen (ROS) and subsequent leakage of lysosomal membranes. These are followed by an increase in intracellular levels of degradative enzymes, the release of neoantigens, and the migration of the calreticulin “eat me” signal to the cell surface.

In contrast to the intrinsic pathway, the extrinsic pathway (sometimes referred to as type II ICD) is able to induce lysosome-induced immunogenic cell death by directly initiating LMP and triggering endoplasmic reticulum stress as a primary effect [56]. The process begins with a burst of reactive oxygen species created outside the cell. This burst can be initiated by a variety of factors, including superoxide-producing enzymes, macrophages, or NK cells [91, 92]. The ROS then reacts with LDL outside the cell to create oxidized LDL (ox-LDL). Uptake through the endocytic pathway of ox-LDL and perhaps other similarly oxidized lipids allows the reactive oxygen species entry into the cytoplasm [93]. This occurs through the specific uptake of modified LDL by scavenger receptors, such as SRAI, SRAII, and CD36 [94, 95]. From here, the cascade of events is almost identical to the intrinsic mechanism where ROS in the cytoplasm degrades the lysosomal membrane and incites the leakage of degradative enzymes into the cell. The caspases and cathepsins trigger ER stress and the superoxide levels in the cell accumulate through increasing mitochondrial membrane dysfunction as well. Calreticulin is shuttled to the cell surface, extracellular ATP is released, and HMGB1 is produced, all markers that immunogenic cell death is occurring. The process of the extrinsic pathway is detailed in Fig. 3. Once calreticulin reaches the cell surface, it can be recognized as an “eat me” signal to dendritic cells which can engulf the dying cell, carry it to the lymph nodes, and present the tumor neoantigens to T cells.

The crucial difference between the extrinsic pathway and the intrinsic is that the extracellular ROS allows for the later bypassing of nuclear involvement—specifically genetic p53—in LIICD. In cancer, where p53 is often mutated, the extrinsic pathway is therefore especially relevant as an inducible mechanism to prime the body’s adaptive immune response against tumor cells.

### The extrinsic pathway and immune surveillance

The external pathway may also be important in immune surveillance. There have been three large placebo-controlled studies (*n* > 15,000 per study) which have shown that use of antioxidants in at-risk populations raised cancer incidence significantly, rather than protecting subjects from cancer as expected. The common mechanism of action in all of these studies shows the potential necessity of ROS in the extracellular environment to induce immunogenic cell death in mutant p53 cancer cell lines. When ROS is decreased with antioxidant therapy, the body’s ability to kill malignant cells also declined.

In the first study, the antioxidants Vitamin E and beta carotene were given to male smokers. There was an 18% increase of cancer incidence in the group receiving antioxidants compared to placebo [96]. In the second study, vitamin A and beta carotene or placebo were given to subjects exposed to asbestos and subjects who were heavy smokers. With 73,137 person-years of follow-up there was a 28% increased incidence of lung cancer in the group receiving antioxidants compared to the placebo group [96]. In the third study, another antioxidant Vitamin E was given to men and an increased incidence of prostate cancer was observed. In a 3-year follow-up, the group receiving antioxidants had a 17% increased incidence of prostate cancer compared to the group receiving placebo which was statistically significant [97].

Early in tumor development, there is a balance between malignant cell growth and the ability of immune surveillance to kill these cells. The addition of antioxidants in the cited studies suppressed one line of immune defense and tipped that balance in favor of malignant cell growth. Antioxidants such as Vitamin E, beta carotene, and Vitamin A interact with and inactivate free radicals. These results strongly suggest that extracellular ROS produced by immune cells play a key role in the natural defense against tumor development.

### Targeting the extrinsic pathway

Several therapies are being developed that use the external pathway as a means to kill cancer cells that were previously thought to be “cold,” that is: unresponsive to traditional immuno-oncology pharmacology.

#### Xanthine oxidase and peroxidases

There are several agents capable of generating ROS in mammalian cells. The most frequently used involve NADH-oxidase, xanthine oxidase, and nitric oxide synthase [98]. It’s been shown that the extent of lysosomal leakage depends directly on oxidase concentration administered [99]. Recent studies have linked xanthine oxidase closely with cancer suppression—demonstrating that XO levels are much lower in tumors of gastrointestinal, breast, lung, kidney, bladder, and ovary tissues compared to normal [100]. Also, lower levels of xanthine oxidase in tumors were linked to worse patient outcomes in ovarian, colorectal, gastric, and breast cancers [101–104].

Due to the preferential cytotoxicity to malignant cells that is induced by peroxidized LDL, xanthine oxidase (a form of xanthine oxidoreductase) and other peroxidases in combination with LDL have been used to trigger LIICD in cancer cells [105]. Studies of malignant mesothelioma cells (JMN-18) and prostate adenocarcinoma (DU-145) treated with p-LDL showed localization of the administered reactive oxygen species to the cancer cells’ lysosomes with subsequent leakage of lysosomal contents and cell death [105]. In experimental rabbit models, hypoxanthine and xanthine oxidase delivered locally significantly suppressed squamous cell carcinoma tumor growth [106]. A recent study in 2019 showed that Alternol, an activator of xanthine oxidase, induces potent oxidation-related damage in prostate cancer cells but not normal cells [98]. These agents are exciting due to their direct induction of LIICD which bypasses the intrinsic pathway and can act on any tumor type, regardless of the cell’s genetic mutations that may have made them resistant to traditional immunotherapies.

#### Hypericin-based PDT

The external pathway of LIICD can be triggered by a type of photodynamic therapy known as hypericin-based PDT (Hyp-PDT). This method is much like the traditional PDT described previously, with the main differentiation being its active photosensitizer. Hypericin localizes predominantly in the endoplasmic reticulum rather than the nucleus. Thus, once a cell is exposed to Hyp, oxygen, and excited with the specific wavelength of visible light, ROS is generated from the ER. This induces subsequent BAX/BAK activation and mitochondrial dysfunction typical of the downstream effects of lysosome-induced apoptosis, as described previously [80]. Hyp-PDT in murine colon cancer and human bladder cancer was shown to cause the hallmarks of immunogenic cell death in pre-apoptotic cells—early exposure of ecto-CALR, extracellular ATP, release of HMGB1, and ecto-HSP70 [88]. Because this therapeutic technique operates on the cell at a more direct level and generates ROS in the cell without nuclear involvement, it is considered an inducer of LIICD via the extrinsic pathway.

#### HSP70 antagonists

Inhibitors of the heat-shock proteins 70, which are stabilizers of the lysosomal membrane proteins, can incite LM permeabilization. Genetic depletion of HSP70 was successfully able to kill glioblastoma, and breast and colon carcinoma cells via LMP [107]. A pharmacological agent 2-phenylethynesulfonamide has also been shown to inhibit heat shock protein 70, and the researchers of this therapy demonstrated evidence of lysosome-induced ICD in primary effusion lymphoma, specifically lysosomal cathepsin D release and activation of dendritic cells to prime an immune response [108]. Similar effects were observed in pancreatic cancer cells [109]. Taken together, pharmacological HSP70 inhibitors are an exciting avenue for inducing LMP without nuclear involvement if their effects can be preferentially observed in tumor cells. More research is needed, however, to verify their selective uptake into cells.

## Perspective and prospective

Achilles was dipped in the river Styx as an infant by his mother Thetis while holding him by the heel. Thus, the only part of Achilles’ body that was vulnerable was his heel. Two recent books, *The Emperor of All Maladies* by Siddhartha Mukherjee and *The Death of Cancer* by Elizabeth DeVita-Raeburn and Vincent T. DeVita document the progress that cancer therapy and cell biology have made over the past half-century. As impressive as that progress has been there is a long way to go. We suggest a focus on the lysosome as a way to strike cancer at its Achilles’ heel. Our body’s immune cells have the potential to not only selectively kill malignant cells, whose changes in their lysosomes already predispose them to LMP, but also become trained to recognize such malignant cells in future presentations to avoid remission or disease entirely. This review details the role of the lysosome in cell death and suggests that therapies which produce ROS in the microenvironment of the tumor may take yet another step to decapitating the Emperor and hastening the Death of Cancer. It may be that when mother Nature dipped cells in the river Styx of evolution, she held them by the lysosome.

## Supplementary information

Author Contributions
