# Supplementary material for: The Achilles’ heel of cancer: targeting tumors via lysosome-induced immunogenic cell death
Source: Cell Death Dis. 2022 May 30;13(5):509. doi: 10.1038/s41419-022-04912-8 (PMC9151667; doi:10.1038/s41419-022-04912-8)
Supplement: Supplementary file 1 — Author Contributions [file 41419_2022_4912_MOESM1_ESM.docx]

Iulianna Taritsa is responsible for the primary drafting, research, and illustrations found in this review.

Dr. Kuldeep Neote serves as an editor for this work and scientific guidance in locating literature.

Dr. Eric Fossel has been a research investigator in the field of immunogenic cell death for over thirty years and has contributed his scientific expertise and guidance in source location and extensive edits.
